# Regulation of Type II Toxin-Antitoxin Systems: The Translation-Responsive Model

**DOI:** 10.3389/fmicb.2020.00895

**Published:** 2020-05-05

**Authors:** Bhaskar Chandra Mohan Ramisetty

**Affiliations:** Molecular Biology and Evolution Lab, School of Chemical and Biotechnology, SASTRA Deemed University, Thanjavur, India

**Keywords:** toxin-antitoxin systems, gene regulation, translation, proteolysis, repression

## Introduction

Most bacterial genomes encode multiple autoregulatory “poison-antidote” gene pairs called Toxin-Antitoxin systems (TAs). They encode a “toxin” that inhibits metabolism and an “antitoxin” that inhibits the activity of toxin protein. TAs are classified into several types, of which Type II TAs are predominantly encoded by bacterial genomes and plasmids (Pandey and Gerdes, 2005). TAs are implicated in a multitude of phenomena such as programmed cell death (PCD), persistence, biofilms, etc., which are proposed to confer eco-evolutionary benefits to the bacteria including pathogens (Hayes and Van Melderen, 2011; Ramisetty and Santhosh, 2017; Harms et al., 2018).

In the recent past, the regulatory mechanisms of TAs and the consequential phenotypes have been debated (Engelberg-Kulka and Glaser, 1999; Hazan et al., 2004; Engelberg-Kulka et al., 2006; Van Melderen, 2010; Hayes and Van Melderen, 2011; Yamaguchi and Inouye, 2011; Ramisetty et al., 2015; Ramisetty and Santhosh, 2017; Harms et al., 2018; Fraikin et al., 2019). Drawing a general model for the vast diversity of Type II TAs is indeed difficult. The variability in the operator architecture, stoichiometries of TA complexes, the activators of TAs and the consequences are diverse. Two models were proposed to explain TAs regulation: “Passive regulation” and “Active regulation” (Gerdes et al., 2005). There is consensus that the concentration of antitoxin relative to the toxin is crucial for the TAs regulation (Chan et al., 2016). But, is the reduction of antitoxin concentration during stress caused by the decrease in antitoxin production or the increase in antitoxin degradation? Here, I delineate the general components and regulators of a typical Type II TAs and then share my opinions on the regulatory models that are specific to Type II TAs.

## Components and Regulators of TAs Circuitry

**(i) Components**: The components of a typical Type II TAs constitute the TA mRNA, antitoxin protein, toxin protein, and TA complex. A typical Type II TA mRNA consists of the two overlapping open reading frames (ORFs); generally the antitoxin ORF is located downstream of the promoter and upstream of the toxin ORF (Gerdes et al., 2005; Harms et al., 2018). Some Type II TAs such as *mqsRA* have an alternate architecture: toxin ORF upstream of antitoxin ORF (Brown et al., 2013). The antitoxin ORF has an optimal Shine-Dalgarno (SD) sequence and AUG start codon while the toxin has suboptimal SD sequence, which is usually a part of the antitoxin ORF. These observations indicate the “differential translation” of TA proteins—the number of antitoxin proteins produced is higher than the number of toxin proteins produced per TA mRNA per unit time. Typically, the antitoxins have loosely folded conformations, which make antitoxins more vulnerable to proteolysis and hence a shorter half-life. On the other hand, toxins are globular and have relatively longer half-life. Hence, the antitoxins and toxins are differentially degraded by the specific proteases, such as Lon and ClpAP (Engelberg-Kulka et al., 1998; Christensen et al., 2001, 2003; Diago-Navarro et al., 2013). The toxin protein interferes in metabolism, via translational or replication inhibition or by disrupting cell membrane integrity (Harms et al., 2018). The antitoxin protein forms a complex with toxin, thereby preventing the toxic effects. The TA complex also functions as the repressor of TAs transcription. The TA complex binds to the operator region upstream of TA genes (negative autoregulation) through the DNA binding motif of the antitoxin protein (Gerdes et al., 2005; Yamaguchi and Inouye, 2011; Chan et al., 2016). Two factors contribute to the dynamism in the binding of TA complex to the operator: (i) the possibility of multiple stoichiometric TA complexes with varying affinities to their respective operators (Monti et al., 2007; Overgaard et al., 2009) and (ii) the architecture of the operator (the number, the orientation and the spacing of the binding sites; Bailey and Hayes, 2009). There seem to be at least two stoichiometric forms of TA complexes; (i) one which neutralizes toxin and (ii) another a transcriptional repressor. The stoichiometry of the TA complex seems to be dependent on toxin-antitoxin affinities and their relative concentrations. Likely, high antitoxin/toxin ratio ([A]/[T]) favors the formation of TA_r_ (repressive complex) while moderate antitoxin/toxin ratio favors the formation of TA_n_ (neutralization complex that cannot bind to the operator). Low antitoxin/toxin ratio discourages TA complex formation. Therefore, the dynamics of the concentration of antitoxin is the central variable in the regulation of TAs.

**(ii) Regulatory processes of the TA circuit**: Cellular metabolic processes, such as transcription, RNA degradation, translation and proteolysis, regulate the TAs genetic circuitry. Transcription rate of TA operon is proportional to the availability of free promoter, which in turn is dependent on the TA_r_ complex binding to the operator. Hence, transcription and translation are the feeders of the TA components. The RNases non-specifically degrade the TA mRNAs. The dilution of TA components also happens due to cell division, which is then replenished to the equilibrium during each cell division. The TA proteins are degraded by specific proteases (Lon protease). The antitoxin is rapidly degraded compared to the toxin: the “differential proteolysis” of TA proteins.

Of the above-mentioned processes, translation and proteolysis of the antitoxins seem to be the key regulators of TAs functioning. Two models were described, “Passive model” and “Active model,” to explain the TAs regulation and function in the general physiology of the bacteria (Gerdes et al., 2005). The two models differ in the regulatory process that causes a decrease in the relative antitoxin concentration during stress. The “Passive model” is based on the suggestion by Hanna Engelberg-Kulka's group that the activation of TAs is caused by the inhibition of the production of antitoxin during stress (Sat et al., 2001). In the “Active regulation” model, the rate of production of the antitoxin is considered to be constant and the rate of proteolysis is thought to be highly variable, i.e., stress triggers antitoxin degradation (Christensen et al., 2003; Gerdes et al., 2005).

## The Controversial Active Model and the Role of Inorganic Polyphosphate

**Figure 1:**
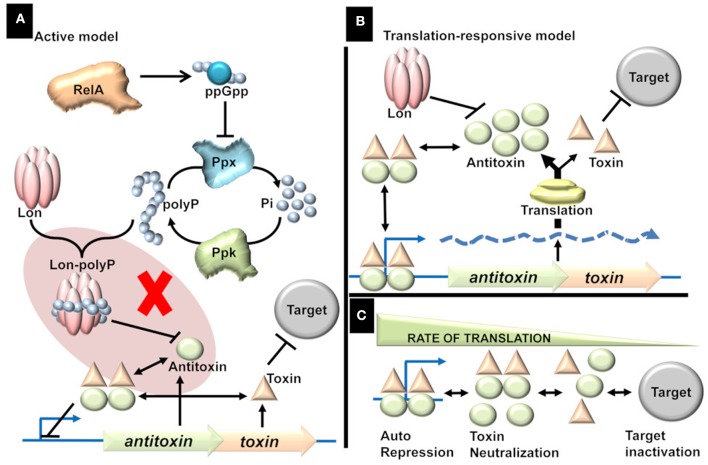
**(A)** Active model. Stress triggers the production of ppGpp, which in turn triggers the accumulation of inorganic polyphosphate (polyP) by inhibiting Exopolyphosphatase (PPX). The accumulation of polyP is proposed to direct Lon protease to specifically target antitoxins thereby activating the TAs. The shaded part represents the flaw in active model [adapted from (Ramisetty et al., 2016)]. There is no evidence to prove that polyP is essential for the Lon dependent degradation of antitoxins. Rather, it was shown that polyP is not involved in the regulation of TAs. **(B)** Translation-responsive model. The concentration of the TA proteins is primarily dependent on two factors; proteolysis by Lon (essential but constant) and translation (highly variable factor). The rate of translation dictates the Antitoxin concentration which in turn influences the TA complex formation and consequently the repression or target inactivation. **(C)** The consequences or state of TAs regulation in response to translation rate. The regulation of TAs ranges from repression (and neutralization of toxin) of the operator to inactivation of the targets; with multiple intermediate states. Higher the translation rate, the higher is the transcriptional repression and lower target inactivation. Lower the translation, lower the transcriptional repression and higher the target inactivation. In intermediate rates of translation, there is incomplete transcriptional repression and incomplete target inactivation.

The Active model, proposed by “Kenn Gerdes” group, is “attractive.” Response to stimuli is elicited in the form of signal molecules which in turn regulate TAs followed by activation of toxins and culmination in a phenotype (Figure 1A). Since the discovery of *relBE* in the studies of stringent response (Mosteller and Kwan, 1976), it was speculated that TAs play a role in stringent response (Christensen and Gerdes, 2004). During amino acid starvation, RelA produces ppGpp, which in turn inhibits the exopolyphosphatase resulting in the accumulation of inorganic polyphosphate (polyP), a key modulator of Lon protease (Rao et al., 1998; Kuroda et al., 2001). It was reported that polyP is essential for the degradation of antitoxins and that the activated toxins induced persistence, a non-inherited multidrug tolerance to lethal doses (Maisonneuve et al., 2013).

Two key points in the Active model were challenged (Osbourne et al., 2014; Ramisetty et al., 2016; Van Melderen and Wood, 2017; Goormaghtigh et al., 2018):(i) the link between polyP and degradation of antitoxins and (ii) induction of persistence by the toxins. The chromosomal TA-mediated persistence was an artifact due to an inadvertent prophage integration within the mutant genome. The papers reporting “evidence” for the active model have now been retracted (Maisonneuve et al., 2018; Retraction, 2018) and follow up studies with a new mutant strain (deleted for ten Type II TAs) ruled out the role of chromosomal TAs in persistence in *E. coli* (Harms et al., 2017; Goormaghtigh et al., 2018). Unfortunately, the role of polyP in TAs regulation was never addressed by the proponents in light of the retractions (Maisonneuve et al., 2018). Does polyP modulate Lon protease to degrade antitoxins? How probable is that all the 10 structurally diverse antitoxins are degraded by Lon protease modulated by polyP?

The Active model predicts that the regulation of TAs is bistable meaning that the TA operon is either “ON” (toxin-dominant state) or “OFF” (antitoxin-dominant state; Cataudella et al., 2013; Fasani and Savageau, 2013). Hence, it is essential to address the direct role of polyP or ppGpp in the regulation as mathematical models are still based on the linear “Active regulation model” (Tian et al., 2017). Firstly, ppGpp is not required for the transcriptional activation of *relBE, chpBS, mazEF*, and *yefM*/*yoeB* (Christensen et al., 2003; Ramisetty et al., 2016). There is transcriptional upregulation as well as toxin dependent mRNA cleavage in mutant strains deficient in accumulation of ppGpp and/or polyP (Ramisetty et al., 2016). Hence, polyP is neither involved in the transcriptional activation of *yefM*/*yoeB* operon nor YoeB dependent target mRNA cleavage. Therefore, the null hypothesis that polyP is not involved in the degradation of antitoxins is accepted, and the “Active regulation model” is rejected.

## The Translation-Responsive Model

The concentration of relative antitoxin is paramount and is influenced by the rate of production (translation) and the rate of degradation (proteolysis). What is causal for the activation of TAs regulation during stress; inhibition of translation or enhancement of proteolysis? The rate of global translation is highly variable. It is a function of the ATP/ADP ratio, amino acid pool, charging of tRNA, production of rRNA, ribosomal proteins, and the formation of functional ribosomes. The advantages of switching off translation are conceivable; the conservation of energy, amino acids, and redirection of translational machinery to produce the stress proteome.

In contrast, the proteolysis is dependent on only two factors; the energy required for proteolysis and the concentration of active proteases available to degrade antitoxins. Upon amino acid starvation, the depletion of charged tRNA causes ribosomal stalling and followed by the activation of proteases to degrade ribosomal proteins (Kuroda et al., 2001). Hence, translational inhibition precedes proteolysis during conditions like amino acid starvation. Hence, it is conceivable that TAs are responsive to translational inhibition rather than the proteolysis of antitoxin. Although I ascribe a lot of significance on the rate of antitoxin production, it is important to note that the proteolytic degradation of antitoxin is essential but constant. For example, in a protease deficient strain the regulation of TAs is “dead” (Christensen et al., 2003; Ramisetty et al., 2016) as there is insignificant degradation of the antitoxin resulting in the production of sufficient TA_r_ and hence constant repression of TA operon. The changes in the transcription of TA operon, which represents the antitoxin/toxin ratio, is a dynamic function of translation and hence this model is referred to as the “Translation-responsive model” (Figure 1B). In the translation-responsive model (based on the “Passive model” Sat et al., 2001), the key regulator of the antitoxins' concentration is the rate of antitoxin production (highly variable) while antitoxin proteolysis is essential but constant.

Experiments involving drastic and instantaneous inhibition of translation, like treatment of exponentially growing *E. coli* with high doses of serine hydroxamate or chloramphenicol, ensure that there is no production of antitoxins but proteolysis continues, and hence a dramatic increase in transcription is observed over time (Christensen et al., 2001; Ramisetty et al., 2016). Based on these observations of TAs transcription upon induction of severe stress, several researchers attribute TAs as bistable systems that are either “ON” (toxin-dominant state or derepressed state) or “OFF” (antitoxin-dominant state or repressed state). The Translation-responsive model predicts that the TAs is rather analog and not be bistable. As opposed to starvation experiments in which TA mRNA seems to accumulate over time, stable maintenance of transcriptional upregulation (~3-fold higher transcription) indicates that the operator is partially repressed. There are a few empirical evidences where an intermediate state (higher than basal level but lower than outright activation) of TAs transcription is observed. In heat shock (47°C) and oxygen deprivation experiments, a consistent three-fold increase in the transcription rate of *yefM/yoeB* loci is maintained throughout the experiment [see Figure 4 of (Ramisetty, 2015)]. These observations indicate that the regulation of *yefM/yoeB* is not just an ON or OFF but has several intermediate states. *relB* mutants (*relB101*) had intermediate transcription compared to the wild type [see Figure 5B of (Bech et al., 1985)]. An intermediate transcription was also observed in the *relBE* system upon heat shock and glucose starvation [see Figure 3A of (Christensen et al., 2001)]. Studies on *relB101* (a single nucleotide polymorphism resulting in A39T mutant of RelB), showed that mutations within the TAs operon could affect the general metabolism. In fact, it was shown that *relB* mutants are sensitive to glucose starvation (Mosteller, 1978). Based on the translation-responsive model we could predict that mutations within the operator, antitoxin gene or toxin gene which may interfere in the antitoxin dimerization, antitoxin interaction with the operator, antitoxin interaction with the toxin, or increase the antitoxins' susceptibility for proteolysis could have drastic effects.

The rate of global translation would determine the concentrations of each component (TA proteins and mRNA). The concentration of the components and the consequent transcriptional repression or target inactivation could be envisioned as a chemical reaction (Figure 1C). When the translation rate is high, the equilibrium is toward effective TA operon repression (OFF state). When the translation rate is low, the equilibrium is toward target inactivation (ON state). In suboptimal conditions, where translation rate is an intermediate state, the repression is incomplete, and there is also a possibility of target inactivation (intermediate state). Alternately, slow-growing pathogenic bacteria (likely to have lower translations rate) such as *Mycobacterium tuberculosis* would be ideal to understand the TAs regulation and the multiplicity of TAs on the general metabolism. The phenotypic heterogeneity in a bacterial population could imply that the translation rates in different bacteria could be different, resulting in a concomitant difference in the TAs expression and phenotypes.

Studies by various groups on different TAs in different model bacteria resulted in the implication of TAs in numerous phenomena such as bacterial programmed cell death, persistence and biofilm formation. However, most of these observations are contended with counter-evidence and debated (Harms et al., 2017; Ramisetty and Santhosh, 2017; Fraikin et al., 2019, 2020). One crucial problem is the lack of complete insights into the TAs regulation. Hence, it is imperative to understand the intricacies of TAs regulation. Further exploration is required to estimate the concentrations of each component at different metabolic states and contexts.

## Conclusions

It could be ambitious to provide one single model for diverse and abundant TAs (Van Melderen and Wood, 2017). The proposed Translation-responsive model is based on the fact that TAs are horizontally transferring genes, which meant that their expression on plasmids and different bacterial genomes must be dependent on general factors such as translation and proteolysis. However, there could be additional factors in the regulation due to proximity of binding of other regulators (other than TA complex) (Uppal and Jawali, 2016) and cross-activation of TAs (Kasari et al., 2013). At the state of current information, the active model of TAs regulation is refuted. The key aspects of the translation-responsive model of Type II TAs regulation are (i) the differential translation and differential proteolysis rates of TA proteins, (ii) antitoxin concentration is highly variable, (iii) translational machinery is the critical regulator, (iv) proteolytic degradation is essential but relatively constant and (v) the TA operon repression by the TA complex is dynamic with the possibility of intermediate states of expression. This model could be applicable to other types of TAs (irrespective of the toxin targets) as long as the principal regulator of the system is under the control of translation. Based on the Translation-responsive model, the physiological significance of TAs could be in natural conditions (suboptimal relative to laboratory conditions) wherein the TA proteins are likely to be in higher concentration or toxins could be relatively active.

## Author Contributions

BR has conceived the idea and wrote the manuscript.

## Conflict of Interest

The author declares that the research was conducted in the absence of any commercial or financial relationships that could be construed as a potential conflict of interest.
